# Mapping QTLs for adult-plant resistance to powdery mildew and stripe rust using a recombinant inbred line population derived from cross Qingxinmai × 041133

**DOI:** 10.3389/fpls.2024.1397274

**Published:** 2024-05-08

**Authors:** Yahui Li, Jinghuang Hu, Huailong Lin, Dan Qiu, Yunfeng Qu, Jiuyuan Du, Lu Hou, Lin Ma, Qiuhong Wu, Zhiyong Liu, Yijun Zhou, Hongjie Li

**Affiliations:** ^1^College of Life and Environmental Science, Minzu University of China, Beijing, China; ^2^The National Engineering Laboratory of Crop Molecular Breeding, Institute of Crop Sciences, Chinese Academy of Agricultural Sciences, Beijing, China; ^3^Jiushenghe Seed Industry Co. Ltd., Changji, China; ^4^Institute of Genetics and Developmental Biology, Chinese Academy of Sciences, Beijing, China; ^5^State Key Laboratory of Crop Stress Adaptation and Improvement, School of Life Sciences, Henan University, Kaifeng, China; ^6^Wheat Research Institute, Gansu Academy of Agricultural Sciences, Lanzhou, China; ^7^Qinghai Academy of Agricultural and Forestry Sciences, Qinghai University/Key Laboratory of Agricultural Integrated Pest Management, Xining, China; ^8^Datong Hui and Tu Autonomous County Agricultural Technology Extension Center, Xining, China; ^9^Institute of Biotechnology, Xianghu Laboratory, Hangzhou, China

**Keywords:** wheat (*Triticum aestivum* L.), powdery mildew, stripe rust, adult-plant resistance, QTL mapping

## Abstract

A recombinant inbred line (RIL) population derived from wheat landrace Qingxinmai and breeding line 041133 exhibited segregation in resistance to powdery mildew and stripe rust in five and three field tests, respectively. A 16K genotyping by target sequencing (GBTS) single-nucleotide polymorphism (SNP) array-based genetic linkage map was used to dissect the quantitative trait loci (QTLs) for disease resistance. Four and seven QTLs were identified for adult-plant resistance (APR) against powdery mildew and stripe rust. *QPm.caas-1B* and *QPm.caas-5A* on chromosomes 1B and 5A were responsible for the APR against powdery mildew in line 041133. *QYr.caas-1B*, *QYr.caas-3B*, *QYr.caas-4B*, *QYr.caas-6B.1*, *QYr.caas-6B.2*, and *QYr.caas-7B* detected on the five B-genome chromosomes of line 041133 conferred its APR to stripe rust. *QPm.caas-1B* and *QYr.caas.1B* were co-localized with the pleiotropic locus *Lr46/Yr29/Sr58/Pm39/Ltn2*. A Kompetitive Allele Specific Polymorphic (KASP) marker *KASP_1B_668028290* was developed to trace *QPm/Yr.caas.1B*. Four lines pyramiding six major disease resistance loci, *PmQ*, *Yr041133*, *QPm/Yr.caas-1B*, *QPm.caas-2B.1*, *QYr.caas-3B*, and *QPm.caas-6B*, were developed. They displayed effective resistance against both powdery mildew and stripe rust at the seedling and adult-plant stages.

## Introduction

Obligate biotrophic fungi are serious pathogens that constantly endanger the production of wheat (*Triticum aestivum* L.) in China, for example, *Blumeria graminis* (DC.) Speer f. sp. *tritici* (*Bgt*) and *Puccinia striiformis* Westend. f. sp. *tritici* Erikss. (*Pst*), inciting wheat powdery mildew and stripe rust, respectively. Powdery mildew is primarily found in wheat-growing areas with maritime climates. It became one of the epidemic diseases since the 1970s, spreading from the southwestern to eastern and northern regions of the country within a few decades. Outbreaks of powdery mildew have affected an average of 7 million hectares of wheat annually from 2002 to 2020 (Xiao et al., 2022). Historically, stripe rust has long been a serious biotic constraint in wheat production and continues to be a yield-limiting disease at present. In 2023, stripe rust occurred on an area of 2.67 million hectares (https://www.natesc.org.cn/).

Assessment of resistance to powdery mildew and stripe rust is a mandated task in national and provincial wheat yield trials before commercializing any cultivars given the importance of these diseases in limiting wheat production in China. The improvement of resistance to these diseases is one of the most important objectives in many wheat breeding programs. Two types of genetic mechanisms conferring disease resistance are available to breeders. One is all-stage resistance (ASR), showing race-specific effectiveness and qualitative inheritance. This type of gene may not be effective when virulent pathotypes of pathogens occur. Genes associated with ASR are characterized by seedling tests with artificial inoculation of a single or a few isolates of pathogens in condition-controlled greenhouse or growth chambers. The other is adult-plant resistance (APR), showing effectiveness at the later growth stage of crops, non-race specific mode, and quantitative inheritance. The loci governing APR are usually characterized in field disease nurseries either artificially inoculated or naturally infected at the adult-plant stage.

Most officially designated powdery mildew (*Pm*) and stripe rust (*Yr*) resistance genes are characterized as ASR genes. Among the designated *Pm* genes, *Pm38* (Spielmeyer et al., 2005), *Pm39* (Lillemo et al., 2008), *Pm46* (Herrera-Foessel et al., 2014), *Pm55* (Zhang et al., 2016), and *Pm62* (Zhang et al., 2018) are identified as the APR genes. Twenty out of 56 *Yr* genes are reported to perform APR to stripe rust. *Yr18* (Singh and Rajaram, 1993), *Yr36* (Uauy et al., 2005), *Yr39* (Lin and Chen, 2007), *Yr52* (Ren et al., 2012), *Yr59* (Zhou et al., 2014), *Yr62* (Lu et al., 2014), and *Yr79* (Feng et al., 2018) are the APR genes against stripe rust. Additionally, 222 and 505 quantitative trait loci (QTLs) for resistance to powdery mildew and stripe rust have been characterized using genetic linkage maps constructed with biparental recombinant inbred line (RIL) populations or genome-wide association study (GWAS) with natural populations (Singh et al., 2022; Kumar et al., 2023).

Wheat diseases caused by different pathogens, such as *Bgt* and *Pst*, often occur concurrently, which complicates the genetic improvement of disease resistance in wheat breeding (Liu et al., 2015). Stacking genes or QTL conferring resistance to different diseases in single genotypes offers a feasible option. For example, winter wheat cultivar Jimai 22 carries *YrJ22* (Chen et al., 2016) and *Pm52* (Qu et al., 2020) for resistance to stripe rust and powdery mildew, respectively. *YrZH84* (Li et al., 2006), *LrZH84* (Zhao et al., 2008), and *QPm.caas-3BS* (Jia et al., 2018) are responsible for resistance to stripe rust, leaf rust, and powdery mildew in winter wheat breeding line Zhou 8425B. *Pm64* for powdery mildew resistance and *Yr5* for stripe rust resistance are tightly linked in wheat-wild emmer (*Triticum turgidum* var. *dicoccoides*) introgression line WE35 (Zhang et al., 2019a). Wheat cultivars or lines resistant to multiple diseases are preferably developed by breeders for broader adaption of cultivars.

We have identified the ASR gene *PmQ* against powdery mildew in wheat landrace Qingxinmai (Li et al., 2020) and the ASR gene *Yr041133* against stripe rust in breeding line 041133 (Li et al., 2022). To stack the resistance to both diseases, we developed a RIL population from the cross of Qingxinmai × 041133. The objectives of this study were to 1) dissect APR loci and 2) develop wheat lines pyramiding loci conferring resistance to both powdery mildew and stripe rust.

## Materials and methods

### Plant materials and field planting

The mapping population of cross Qingxinmai × 041133 comprised 228 F_2:9_ RILs. Wheat landrace Qingxinmai from Xinjiang carries gene *PmQ* on the chromosome arm 2BL for its resistance to powdery mildew at the seedling stage (Li et al., 2020). Wheat breeding line 041133 (pedigree: Jining 13 × Tongmai 2) from Qinghai Province carries gene *Yr041133* on the chromosome arm 7BL for its seedling resistance tests to stripe rust (Li et al., 2022). Field disease nurseries were established at Beijing (BJ; 116.33°E 39.96°N) and Changping, Beijing (CP; 116.26°E 40.17°N) for assessing resistance to powdery mildew and Qingshui, Gansu Province (QS; 105.80°E 34.60°N) for assessing resistance to stripe rust at the adult-plant stage (Hu et al., 2023). All the field trials were carried out using a randomized complete block design with two replicates. Each plot consisted of a single row 1 m in length in sites BJ and CP and 1.5 m in length in QS. Approximately 40–50 seeds were sown in each row.

### Assessments of APR and ASR to powdery mildew

Field trials were carried out to assess powdery mildew resistance at the adult-plant stage at the CP site in 2018–2019 (2019CP), 2019–2020 (2020CP), and 2020–2021 (2021CP) and the BJ site in 2021–2022 (2022BJ) and 2022–2023 (2023BJ). Plants of susceptible wheat spreader, Zhongzuo 9504, were grown every 20 rows and around the experimental plots. They were inoculated with a mixture of isolates designated *Bgt27* at the jointing stage [growth stage (GS) 26] (Zadoks et al., 1974). *Bgt27* was produced by mixing prevalent isolates E09, E15, E21, E23-(2), and E31, and it was virulent on 20 known *Pm* genes (Wang et al., 2022; Hu et al., 2023). Maximum disease severity (MDS) on penultimate leaves was rated as the percentage of leaf area covered by *Bgt* colonies at the late grain-filling stage (GS 77) (Lan et al., 2009). The mean MDS value from five plants randomly selected in each plot was calculated to represent the phenotype of each entry.

The seedling test for assessing powdery mildew resistance was carried out twice following a previously described method (Li et al., 2020). Ten seedlings per line at two-leaf-stage were inoculated with isolates *Bgt27* and *Bgt1* collected from Yuncheng, Shandong Province. After incubation in a dew plastic bag for 24 h, inoculated seedlings were grown in a greenhouse set at 15°C–18°C to allow symptom development for 2 weeks. Infection types (ITs) on primary leaves were rated on a 0–4 scale. Plants with ITs 0 (immune), 0; (hypersensitive reaction), 1 (highly resistant), and 2 (moderately) were categorized into the resistant group, and those with ITs 3 (moderately susceptible) and 4 (highly susceptible) were classified into the susceptible group.

### Assessments of APR and ASR to stripe rust

The adult-plant resistance to stripe rust was assessed in the field disease nursery set at the QS site in 2018–2019 (2019QS), 2019–2020 (2020QS), and 2020–2021 (2021QS). Spreader plants of Huixianhong were planted every 30 rows and around the plots and inoculated by spraying with urediniospores of a mixture of *Pst* races *CYR32*, *CYR33*, and *CYR34* at GS 26 (Bai et al., 2022). At GS 77 when the susceptible control Huixianhong was fully infected, disease severity (DS) for the RILs and their parents were scored on the modified Cobb’s scale as described by Peterson et al. (1948).

The *Pst* race *CYR34* was used to perform the seedling resistance test to stripe rust as described previously (Li et al., 2022). Briefly, wheat seedlings at the two-leaf stage were inoculated with *Pst* urediniospores suspended in light mineral oil (Novec 7200) at 4 mg/mL. After incubation at 9°C–13°C for 24 h in a dew chamber, inoculated plants were grown in a greenhouse for symptom development. A previously established 0–9 scale was used to rate the ITs on primary leaves (McNeal et al., 1971).

### Genetic map construction and QTL detection

A genetic linkage map was previously constructed using the same RIL population as described in another report (Li et al., 2024). The RIL population and the parents were genotyped using the wheat 16K genotyping by target sequencing (GBTS) single-nucleotide polymorphism (SNP) array (MolBreeding Biotechnology Co. Ltd., Shijiazhuang, China, http://www.molbreeding.com). A genetic linkage map (3,113.1 cM in length) was constructed using 2,398 bin SNP markers polymorphic between parents Qingxinmai and line 041133. Analysis of QTLs for resistance to powdery mildew and stripe rust was performed using IcIMapping 4.2 software (Meng et al., 2015). The QTLs repeatedly detected in at least two environments and the best linear unbiased estimate (BLUE) datasets were considered the stable QTLs. The physical positions of the QTLs identified were determined by projecting the closely linked markers on the Chinese Spring reference genome sequence RefSeq v1.0 (IWGSC, 2018). MapChart v2.3 software was used to draw the genetic maps for the regions where stable QTLs reside (Voorrips, 2002).

### Development of KASP markers

The SNP marker closely linked to the QTLs on chromosome 1BL was converted to a Kompetitive Allele Specific Polymorphic (KASP) marker. Primer was designed using the web-based tool Polymarker (http://www.polymarker.info/). The reaction mixture (10 µL) was generated by mixing 4 µL genomic DNA (50 ng/µL), 5 µL 2× KASP master mix, 0.7 µL primer mix (12 mM of each allele-specific primer and 30 mM of the common primer), and 0.3 µL ddH_2_O. The following profile for DNA amplification was run in the ABI 7500 device (Applied Biosystems, Foster City, CA, USA): initial denaturation at 94°C for 15 min, 35 cycles of 94°C for 20 s, and 60°C for 1 min. Blue (521 nm) and red (556 nm) fluorescent signals were read at 25°C for 2 min in a FLUOstar Omega microplate reader (BMG Labtech, Durham, NC, USA). They were transformed into FAM homozygote, HEX homozygote, and FAM/HEX heterozygote genotypes with Klustering Caller software (http://www.lgcgroup.com/).

### Prediction of candidate genes

Genes within the mapping interval of the QTLs on chromosome 1BL were extracted from the Chinese Spring reference genome sequence RefSeq v1.0 annotations (https://wheat-urgi.versailles.inra.fr/). Spatiotemporal expression patterns of candidate genes were analyzed in the Wheat Expression Browser (http://www.wheat-expression.com/).

### Statistical analysis

The BLUE and the broad-sense heritability (*H*^2^) were calculated using the Aov (ANOVA of multi-environmental trials) function in the QTL IciMapping software (Meng et al., 2015). Phenotypic correlations and frequency distributions were computed from different environments and the BLUE value of each line in SPSS v. 20.0 for Windows (IBM SPSS, Armonk, NY, USA).

## Results

### Phenotypic performances of APR against powdery mildew and stripe rust

**Figure 1:**
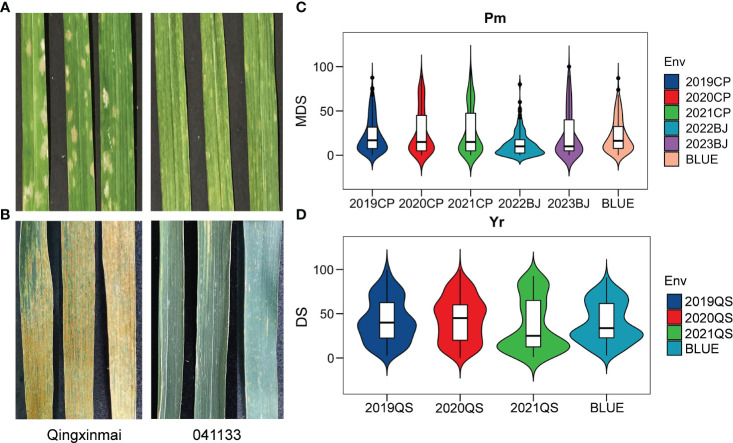
Phenotypes of parents Qingxinmai and line 041133 and their RIL population to *Blumeria graminis* f. sp. *tritici* isolate mixture *Bgt27* and *Puccinia striiformis* f. sp. *tritici* race mixture (*CYR32*, *CYR33*, and *CYR34*) at the adult-plant stage. **(A, B)** The powdery mildew-infected **(A)** and stripe rust-infected **(B)** penultimate leaves of Qingxinmai and line 041133 sampled at the grain-filling stage when disease severity was scored. **(C, D)** Disease scores of powdery mildew **(C)** and stripe rust **(D)** of the RIL population obtained in the field tests at the disease nurseries set at sites Changping (CP), Beijing (BJ), and Qingshui (QS). BLUE, best linear unbiased estimate; RIL, recombinant inbred line.

Reactions of the Qingxinmai × 041133 RIL population and the parents to isolate *Bgt27* at the adult-plant stage were assessed in five field trials conducted in two farms at Changping and Beijing during the wheat growing seasons of 2019–2023. The mean MDS of line 041133 (4.62% ± 4.04%) across sites and years was significantly smaller than that of Qingxinmai (40% ± 4.47%) (*p* < 0.05) (**Figure 1A**). Values of MDS for the RILs displayed a continuous distribution in a range of 0% to 100% with the coefficients of variation (CVs) from 0.84 to 1.11 in different trials (**Table 1**, **Figure 1C**). Frequency distributions of MDS for the RILs were nearly normal in all environments, except for 2022BJ. This was demonstrated by the absolute values of the Skewness and Kurtosis coefficients (**Table 1**). A high value of broad-sense heritability (*H*^2^ = 0.94) across environments was observed for the APR against powdery mildew.

**Table 1:** Phenotypic variation of powdery mildew and stripe rust of the parents and the RIL population in different environments.

Field test	Parents		RILs				
	Qingxinmai	041133	Range	Kurtosis	Skewness	CV (%)	*H*^2^
Powdery mildew							
2019CP	40	0	0–87.50	0.24	1.03	0.85	0.94
2020CP	35	0	0–90.00	−0.47	0.91	0.94	
2021CP	45	10	0–100.00	−0.49	0.88	0.96	
2022BJ	35	5	0–80.00	4.92	1.91	1.11	
2023BJ	45	5	0–100.00	0.27	1.19	1.09	
BLUE	40	7.74	0–86.96	0.22	1.00	0.84	
Stripe rust							
2019QS	70	0	5–100.00	−0.84	0.34	0.77	0.72
2020QS	60	0	0–100.00	−1.04	0.07	0.60	
2021QS	80	5	1–92.50	−1.24	0.53	0.80	
BLUE	75	2.52	2.75–90.74	−1.09	0.32	0.56	

CP, BJ, and QS indicate field disease nurseries at sites Changping, Beijing, and Qingshui, respectively.

CV, coefficient of variation; H^2^, broad-sense heritability; BLUE, best linear unbiased estimate.

The RIL population of cross Qingxinmai × 041133 was grown in the field disease nursery established at site QS to assess their reactions to stripe rust during the three wheat growing seasons of 2019–2021. Line 041133 produced a mean DS value of 1.88% ± 2.40%, which was significantly smaller than that of Qingxinmai (71.25% ± 8.54%) (*p* < 0.05) (**Figure 1B**). The DS values of the RILs across different years ranged from 0% to 100% with the CVs of 0.56–0.80 (**Figure 1D**). Frequency distributions of DS values for the RILs were nearly normal across years and the BLUE dataset demonstrated by the absolute values of the Skewness and Kurtosis coefficients (**Table 1**). The *H*^2^ of stripe rust resistance was 0.72.

**Figure 2:**
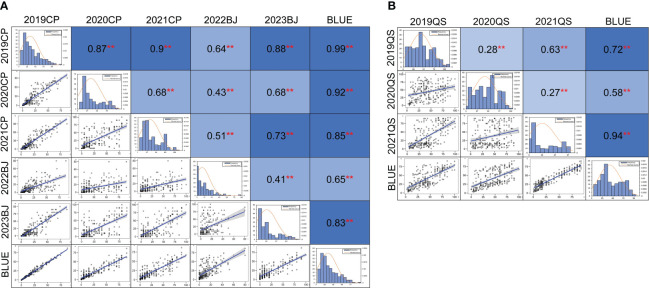
Phenotypic distribution and correlation coefficients of powdery mildew **(A)** and stripe rust **(B)** in the Qingxinmai × 041133 RIL population based on the best linear unbiased estimate (BLUE) datasets. **Significant at *p <* 0.01. The field tests at the disease nurseries set at Changping (CP), Beijing (BJ), and Qingshui (QS) sites. BLUE, best linear unbiased estimate; RIL, recombinant inbred line.

A significant correlation of MDS for powdery mildew was observed among the five field trials and the BLUE dataset with a range of Pearson’s correlation coefficients from 0.41 to 0.99 (*p* < 0.01) (**Figure 2A**). Pearson’s correlation coefficients for the stripe rust DS values across different years ranged from 0.27 to 0.94 (*p* < 0.01) (**Figure 2B**). Significant correlations were also observed among most of the disease rating scores for powdery mildew and stripe rust (**Supplementary Table 1**).

### QTL detection

Four and seven QTLs for APR against powdery mildew and stripe rust were identified, respectively (**Table 2**). Five of them, located on chromosomes 1B, 2B, 3B, and 6B, were stably detected in at least two field trials and the BLUE datasets, explaining 3.73%–20.48% of the phenotypic variations.

**Table 2:** Quantitative trait loci (QTLs) for powdery mildew and stripe rust identified from different environments in the Qingxinmai × 041133 RIL population.

QTLs	Environments	Chromosome	Interval (cM)	Flanking markers	LOD	PVE (%)	Add
Powdery mildew							
*QPm.caas-1B*	2019CP	1B	175.6–179.2	1B_668028290–1B_670413689	11.90	18.67	−9.08
	2020CP	1B	175.6–179.2	1B_668028290–1B_670413689	10.12	18.43	−11.05
	2021CP	1B	175.6–179.2	1B_668028290–1B_670413689	7.43	11.61	−10.08
	2022BJ	1B	175.6–179.2	1B_668028290–1B_670413689	9.28	13.45	−5.44
	2023BJ	1B	175.6–179.2	1B_668028290–1B_670413689	6.94	11.74	−9.61
	BLUE	1B	175.6–179.2	1B_668028290–1B_670413689	13.14	20.48	−9.14
*QPm.caas-2B.1*	2019CP	2B	108.7–109.8	2B_564875204–2B_575171571	3.72	5.13	4.84
	2021CP	2B	108.7–109.8	2B_564875204–2B_575171571	2.61	3.73	5.80
	2023BJ	2B	108.7–109.8	2B_564875204–2B_575171571	2.64	4.04	5.74
	BLUE	2B	108.7–109.8	2B_564875204–2B_575171571	3.68	4.88	4.56
*QPm.caas-2B.2*	2022BJ	2B	123.5–124.5	2B_712772147–2B_737862890	11.17	15.63	5.85
*QPm.caas-5A*	2021CP	5A	82.5–97.5	5A_470185452–5A_504711920	2.85	5.04	−6.61
Stripe rust							
*QYr.caas-1B*	2019QS	1B	175.6–179.2	1B_668028290–1B_670413689	3.96	6.98	−6.02
	2021QS	1B	175.6–179.2	1B_668028290–1B_670413689	10.36	14.11	−11.24
	BLUE	1B	175.6–179.2	1B_668028290–1B_670413689	8.11	11.67	−7.71
*QYr.caas-2D*	2021QS	2D	1.95–12.2	2D_11196322–2D_19623089	3.96	5.99	7.31
	BLUE	2D	1.9–12.2	2D_11196322–2D_19623089	2.84	4.52	4.78
*QYr.caas-3B*	2019QS	3B	0–1.9	3B_6250595–3B_6760906	2.66	4.58	−4.89
	2021QS	3B	0–1.9	3B_6250595–3B_6760906	10.51	13.36	−10.95
	BLUE	3B	0–1.9	3B_6250595–3B_6760906	8.83	12.11	−7.85
*QYr.caas-4B*	2019QS	4B	139.0–143.9	4B_624942478–4B_638364384	4.95	10.76	−7.49
*QYr.caas-6B.1*	2019QS	6B	20.8–31.1	6B_41379534–6B_135142003	3.42	5.98	−5.61
*QYr.caas-6B.2*	2020QS	6B	138.0–138.2	6B_576019211–6B_576246669	4.18	8.55	−7.34
	2021QS	6B	138.0–138.2	6B_576019211–6B_576246669	4.89	5.87	−7.52
	BLUE	6B	138.0–138.2	6B_576019211–6B_576246669	6.82	9.17	−7.08
*QYr.caas-7B*	2021QS	7B	57.5–59.5	7B_145829422–7B_337209575	4.59	5.60	−7.07
	BLUE	7B	57.5–59.5	7B_145829422–7B_337209575	3.27	4.26	−4.66

Positive values indicate that alleles from Qingxinmai increase the trait scores, and negative values indicate that alleles from line 041133 increase the trait scores. CP, BJ, and QS indicate field disease nurseries at sites Changping, Beijing, and Qingshui, respectively.

PVE, phenotypic variation explained; LOD, logarithm of the odds; Add, additive effect; BLUE, best linear unbiased estimate.

#### QTLs for powdery mildew resistance

**Figure 3:**
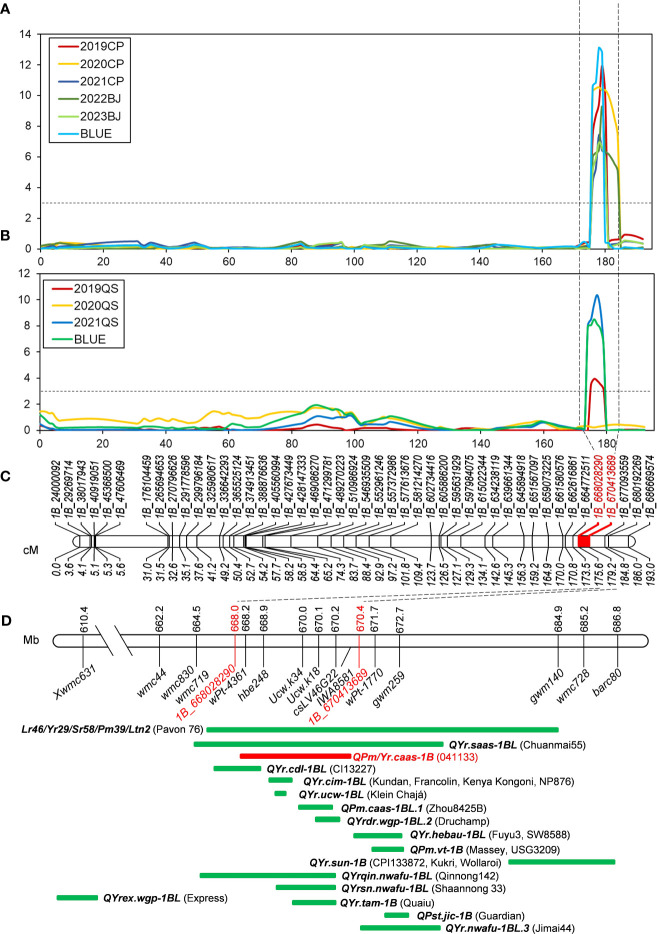
QTL mapping of *QPm/Yr.caas-1B*. *QPm/Yr.caas-1B* for resistance to powdery mildew **(A)** and stripe rust **(B)** in the Qingxinmai × 041133 RIL population at the logarithm of the odds (LOD) of 3.0. **(C)** Linkage map of chromosome 1BL constructed using SNP markers generated by the 16K GBTS SNP array. **(D)** Comparison of *QYr.caas-3B* (red bar) identified in this study and genes/QTLs (green bars) previously mapped on chromosome 1BL for resistance to powdery mildew and stripe rust based on the physical positions of linked molecular markers projected in the Chinese Spring reference genome RefSeq v1.0. QTL, quantitative trait locus; RIL, recombinant inbred line; SNP, single-nucleotide polymorphism; GBTS, genotyping by target sequencing.

Two stable QTLs for powdery mildew resistance were identified on chromosomes 1B and 2B. *QPm.caas-1B* was detected in all five field trials and the BLUE dataset. It explained 11.61%–20.48% of the phenotypic variation with a range of logarithm of the odds (LOD) values from 6.94 to 13.14 (**Table 2**, **Figure 3A**). The resistance allele of these QTLs was derived from line 041133. *QPm.caas-2B.1* was detected in three field trials, 2019CP, 2021CP, and 2023BJ, and the BLUE datasets, showing minor effects by explaining 3.73%–5.13% of the phenotypic variations (LOD = 2.61–3.72) (**Table 2**; **Supplementary Figure 1A**). The resistance allele of this locus was contributed by Qingxinmai. *QPm.caas-2B.2* and *QPm.caas-5A* were detected in only one environment, explaining 15.63% (LOD = 11.17) and 5.04% (LOD = 2.85) of the phenotypic variations, respectively. The positive alleles were contributed by Qingxinmai and line 041133, respectively (**Table 2**).

#### QTLs for stripe rust resistance

Five stable QTLs for APR against stripe rust were detected on chromosomes 1B, 2D, 3B, 6B, and 7B. Three of them, *QYr.caas-1B*, *QYr.caas-3B*, and *QYr.caas-6B.2*, were detected in two environments and the BLUE datasets, explaining 6.98%–14.11%, 4.58%–13.36%, and 5.87%–9.17% of the phenotypic variations, respectively (**Table 2**). The LOD values for these loci ranged from 2.66 to 10.51 (**Table 2**, **Figure 3B**; **Supplementary Figures 2A**, **3A**). Another two QTLs, *QYr.caas-2D* and *QYr.caas-7B* explaining 4.52%–5.99% and 4.26%–5.60% of the phenotypic variations, respectively, were detected in single environments and the BLUE dataset. The resistance alleles of all the stable QTLs were contributed by line 041133, except for *QYr.caas-2D*, which was contributed by Qingxinmai. The remaining two loci, *QYr.caas-4B* and *QYr.caas-6B.1*, were detected in single environments, accounting for 5.98%–10.76% of the phenotypic variations. The resistance alleles of both loci were contributed by line 041133 (**Table 2**).

### Develop KASP marker tightly linked to *QPm/Yr.caas-1B*

The DNA sequences flanking the SNP marker *1B_668028290* obtained from the CS reference genome RefSeq v1.0 were used to design the KASP marker, *KASP_1B_668028290*, linked to *QPm/Yr.caas-1B* (**Supplementary Table 2**). This KASP marker was verified by genotyping the complete RIL population (**Supplementary Figure 4**). As expected, lines with the positive allele derived from 041133 produced significantly smaller disease severity scores of powdery mildew and stripe rust than those with the negative allele in all environments and the BLUE datasets, except for 2020QS (*P* < 0.01) (**Supplementary Figure 5**).

### Effects of major QTLs on disease resistance

**Figure 4:**
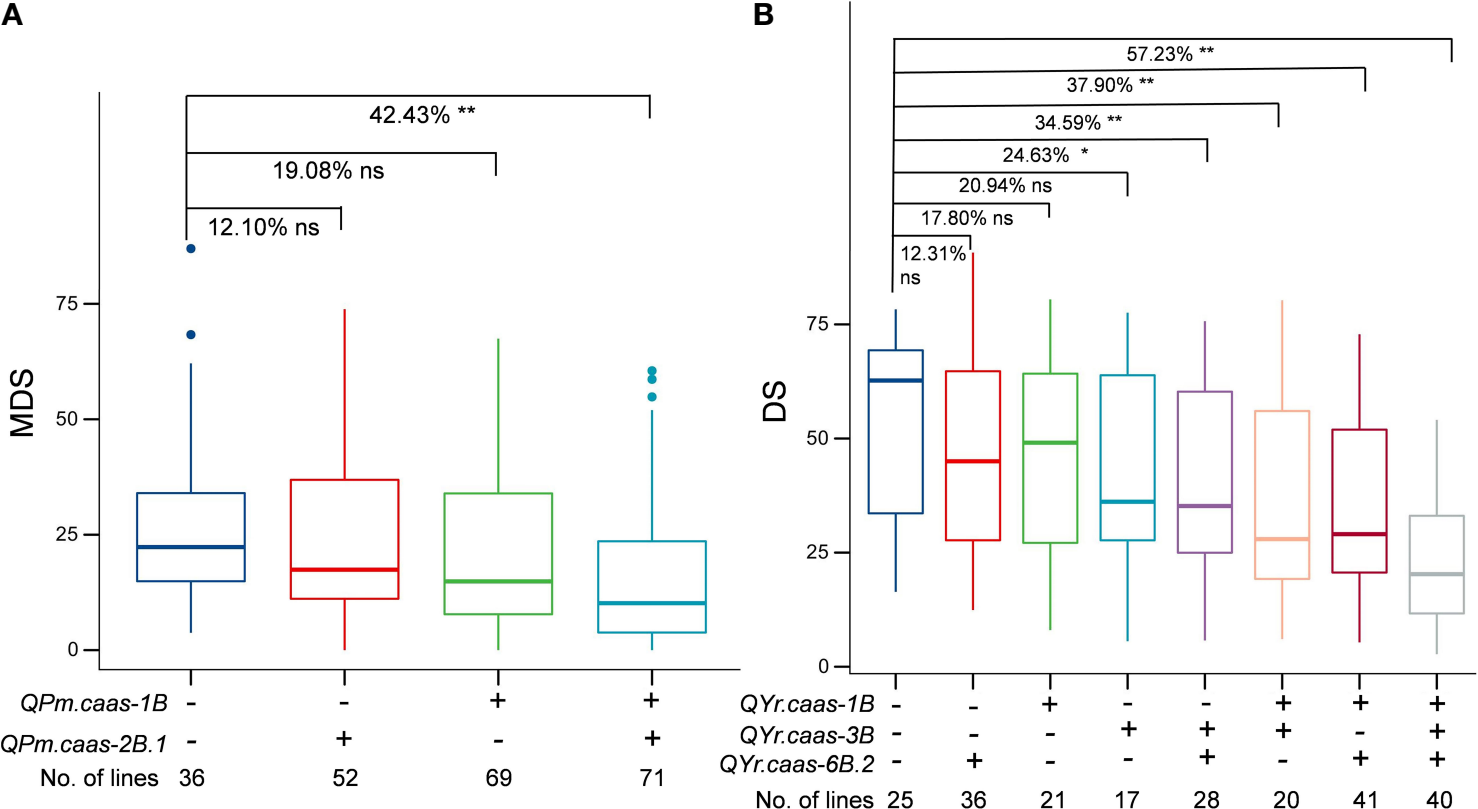
Additive effects of powdery mildew-related QTLs **(A)** and stripe rust-related QTLs **(B)** in the Qingxinmai × 041133 RIL population. + and − indicate lines with and without the positive alleles of the target QTLs based on the flanking markers of the corresponding QTLs, respectively. QTLs, quantitative trait loci; RIL, recombinant inbred line. ns: not significant, *: significant at P < 0.05, **: significant at P < 0.01.

We analyzed the effects of major QTLs, *QPm.caas-1B*, *QPm.caas-2B.1*, *QYr.caas-1B*, *QYr.caas-3B*, and *QYr.caas-6B.2*, on their resistance to powdery mildew and stripe rust by comparing the BLUE datasets of disease scores observed in the RIL population. Lines with *QPm.caas-1B* and *QPm.caas-2B.1* alleles significantly reduced MDS values of powdery mildew (*p* < 0.01) (**Figure 4A**). The stripe rust resistance appeared to be associated with the number of QTLs in the RILs. Compared to the lines that were free of any of QTLs *QYr.caas-1B*, *QYr.caas-3B*, and *QYr.caas-6B.2*, lines with two and three alleles significantly reduced the DS values of stripe rust (*p* < 0.01) (**Figure 4B**).

### Pyramiding of the loci conferring powdery mildew and stripe rust resistance

Based on the genotypes of the molecular markers linked to the two ASR genes, *PmQ* and *Yr041133*, and four APR loci, *QPm/Yr.caas-1B*, *QPm.caas-2B.1*, *QPm.caas-6B*, and *QYr.caas-3B*, four lines pyramiding all loci and one line without any loci were selected. The four pyramided lines QH18, QH19, QH82, and QH202 were resistant to the mixtures of *Bgt* isolates (*Bgt27*) and *Pst* races (*CYR32*, *CYR33*, and *CYR34*) at the adult-plant plant stage with the mean disease scores of 1.7%–8.7% and 2.8%–11.0%, respectively (**Supplementary Table 3**). These lines were also resistant to *Bgt1* and *CYR34* pathogens at the seedling stage showing ITs 0, 0; or 1. However, line QH102, which was free of any resistance locus, was susceptible to any of these pathogens at the adult-plant stage (mean disease scores of 68.3% and 69.9%) and the seedling stage (ITs 4 and 3) in the powdery mildew and stripe rust tests. Qingxinmai was susceptible to isolate *Bgt27* at both the adult-plant and seedling stages. It was also susceptible to the *Pst* race *CYR34* at both growth stages. Line 041133 was susceptible at the seedling stage but resistant at the adult-plant stage to *Bgt27* (**Supplementary Table 3**).

### Potential candidate genes of *QPm/Yr.caas-1B*

**Figure 5:**
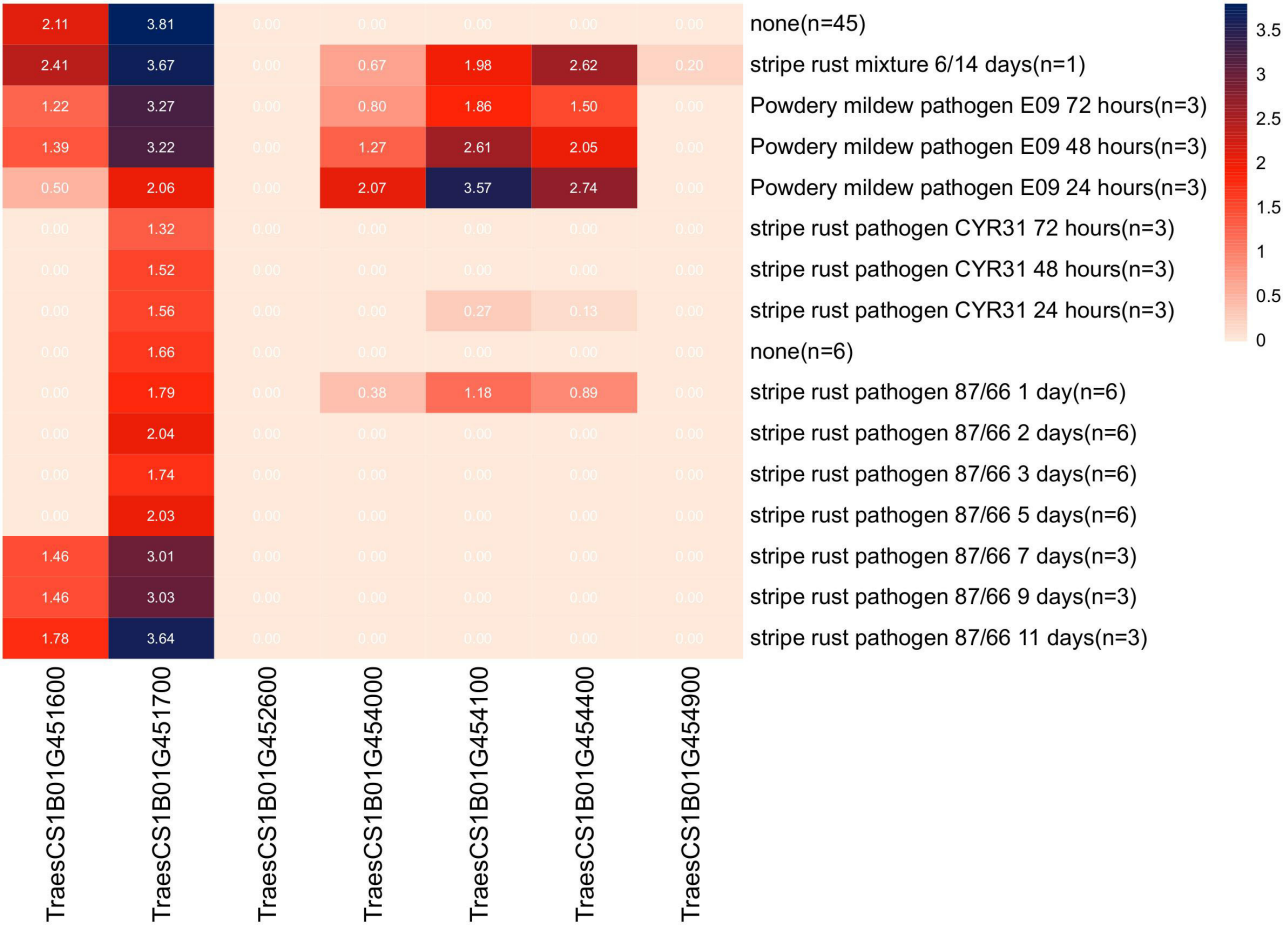
Expression patterns of seven candidate disease resistance genes annotated in the genomic interval of locus *QPm/Yr.caas-1B* in the Wheat Expression Browser (http://www.wheat-expression.com/).

Sequence alignment of the *QPm/Yr.caas-1B*-flanking markers *1B_668028290* and *1B_670413689* resulted in a corresponding physical interval of 2.38 Mb (668.03–670.41 Mb) in the chromosome arm 1BL of Chinese Spring reference genome sequence RefSeq v1.0 (**Table 2**, **Figure 3**). Seven genes for disease resistance were annotated in this genomic interval, including a CC-NBS-LRR (CNL) and six protein kinases (**Supplementary Table 4**). The expression patterns of these disease resistance genes were analyzed using the publicly available database at Wheat Expression Browser (http://www.wheat-expression.com/) (**Figure 5**). The expression of *TraesCS1B01G451600* was induced by *Bgt* isolate E09, but it was not expressed until 7 d post inoculation (dpi) of the *Pst* race *CYR31*. A varying degree of expression was observed for gene *TraesCS1B01G451700* after inoculation with both *Bgt* and *Pst* pathogens. The expression levels of genes *TraesCS1B01G454000*, *TraesCS1B01G454100*, and *TraesCS1B01G454400* peaked at 24 h after inoculation with *Bgt* isolate E09. Neither *Bgt* nor *Pst* was able to induce the expression of *TraesCS1B01G452600* (CNL) and *TraesCS1B01G454900*.

Previously, gene *TraesCS1B01G454100* encoding a receptor-like protein kinase (RLK) was regarded as one of the candidate genes of *QYr.ucw-1BL* (Cobo et al., 2019), which shares a similar genetic interval as *QPm/Yr.caas.1B* (**Figure 3D**). However, the flanking markers of these QTLs, *ucw.k34* and *ucw.k18*, exhibited monomorphism between Qingxinmai and line 041133 (data not shown). The predicted open reading frame (ORF) sequence of *TraesCS1B01G454100* was also identical between Qingxinmai and line 041133 (**Supplementary Figure 6**). This indicates that it is most likely not the candidate gene for *QPm/Yr.caas-1B*. Based on these findings, *TraesCS1B01G451600*, *TraesCS1B01G451700*, and *TraesCS1B01G454000* are possible candidate genes for *QPm/Yr.caas.1B*, which warrants further functional verification.

## Discussion

### Genetic control of powdery mildew and stripe rust resistance in line 041133

The seedling resistance of line 041133 to *Pst* race *CYR34* was controlled by the ASR gene *Yr041133* on chromosome 7BL (Li et al., 2022). The current study observed the adult-plant resistance to a mixture of *Pst* races *CYR32*, *CYR33*, and *CYR34* in this breeding line. The stripe rust resistance in line 041133 was governed by seven QTLs, *QYr.caas-1B*, *QYr.caas-3B*, *QYr.caas.6B.2*, *QYr.caas-2D*, *QYr.caas-4B*, *QYr.caas.6B.1*, and *QYr.caas-7B*, with the first three loci repeatedly detected in multiple environments. Although *QYr.caas-7B* was observed on chromosome 7B (145.83–337.20 Mb), it appeared not to overlap with *Yr041133* (608.90–609.70 Mb). None of the QTLs were located on the same chromosome region as the ASR gene *Yr041133*.

Line 041133 was susceptible at the seedling stage to *Bgt27* and another 23 *Bgt* isolates tested in a previous study (Li et al., 2020). However, it was resistant to powdery mildew at the adult-plant stage when tested with isolate *Bgt27*. Results of QTL mapping detected *QPm.caas-1B* on chromosome 1B and *QPm.caas-5A* on chromosome 5A of line 041133, which conferred its adult-plant resistance against powdery mildew.

### Genetic control of powdery mildew in wheat landrace Qingxinmai

Qingxinmai was previously characterized to be resistant to isolate *Bgt1* due to the presence of the ASR gene *PmQ* on the chromosome arm 2BL (Li et al., 2020). The *Bgt* isolate mixture *Bgt27* was not suitable to determine the effect of *PmQ* on its adult-plant resistance to powdery mildew, as Qingxinmai was not as effective against powdery mildew at the adult-plant stage as that at the seedling stage. However, we detected two minor QTLs, *QPm.caas-2B.1* and *QPm.caas-2B.2*, on the chromosome arm 2BL of Qingxinmai. *QPm.caas-2B.1* contributes 3.73% to 5.13% of phenotypic variations for reducing MDS in three environments. Locus *QPm.caas-2B* in Japanese cultivar Fukuhokomugi (Liang et al., 2006), locus *QPm.inra-2B* in French breeding line RE9001 (Bougot et al., 2006), locus *QPm.vt-2B* in US cultivars Massey (Liu et al., 2001), and locus *Pm.vt-2BL* in USG3209 (Tucker et al., 2007) did not overlap with the mapping interval of *QPm.caas-2B.1*. The physical positions of *QPm.caas-2BL* (546.94–685.76 Mb) in Lumai 21 (Lan et al., 2010b) and *QPmtj.caas-2BL* (568.44–706.97 Mb) in breeding line Tianmin 668 (Hu et al., 2023) coincided with those of *QPm.caas-2B.1*. However, no pedigree connection was recorded between Lumai 21, Tianmin 668, and Qingxinmai. Another QTL, *QPm.caas-2B.2*, is likely identical to *PmQ*, as they share the same genomic interval on the chromosome arm 2BL of Qingxinmai. Since Qingxinmai was not effective against isolate *Bgt27* at both the seedling and adult-plant stages, we were not able to accurately determine the effectiveness of *PmQ* on the adult-plant resistance using isolate *Bgt27* and the Qingxinmai × 041133 RIL population in the current study.

### Pleiotropic locus for resistance to stripe rust and powdery mildew on chromosome 1BL of line 041133

*QPm.caas-1B* and *QYr.caas-1B* for the adult-plant resistance of line 041133 to powdery mildew and stripe rust were co-localized in the same genetic interval on the chromosome arm 1BL. Correlations between the disease severity scores of powdery mildew and stripe rust measured in the Qingxinmai × 041133 RIL population demonstrated that there may exist a common genetic mechanism conferring the resistance to the two diseases (Lillemo et al., 2008).

The chromosomal region where *QPm/Yr.caas-1B* was detected appears to be rich in genetic loci for resistance to wheat powdery mildew and stripe rust. A well-characterized locus *Lr46/Yr29/Sr58/Pm39/Ltn2*, conferring multiple fungal pathogens and leaf tip necrosis, was identified in this genomic region in Mexican spring wheat cultivar Pavon 76 and several other wheat cultivars from different countries (Li et al., 2014). For example, the first designated gene in this region is *Lr46* for leaf rust resistance (Singh et al., 1998). A gene that is closely linked to *Lr46* was characterized as *Yr29* for resistance to stripe rust (William et al., 2003). Gene *Pm39* was detected at the locus *Lr46/Yr29* in a CIMMYT breeding line Saar (Lillemo et al., 2008). The leaf tip necrosis gene *Ltn2* was co-segregated with these rust and powdery mildew resistance genes (Rosewarne et al., 2006), which can serve as a phenotypic marker trait for these disease resistance genes.

There are also some other sources of locus *Lr46/Yr29* from different countries. For example, the Uruguayan wheat cultivar Americano 25e proved to carry *Lr46*, which was derived from a landrace (Kohli, 1986; Kolmer, 2015). Locus *Yr29/Lr46* was detected in Indian cultivars New Pusa 876 and Sujata (Lan et al., 2015; Ponce-Molina et al., 2018a). Chuanmai 55 selected from the cross SW3243 × SW8688 carries *QYr.saas-1BL* co-localized with *Yr29*/*Lr46* (Yang et al., 2019). Additionally, the QTLs for stripe rust resistance detected in Fuyu 3 (Gebrewahid et al., 2020), SW8588 (Zhang et al., 2019b), Qinnong 142 (Zeng et al., 2019), Shaannong 33 (Huang et al., 2021), and Jimai 44 (Liu et al., 2023) overlapped with *Yr29*/*Lr46*. However, it is still not determined with certainty whether this multi-pathogen resistance locus is caused by the pleiotropic effects of a single gene or multiple-linked genes (Cobo et al., 2019).

In addition to *Lr46/Yr29/Sr58/Pm39/Ltn2*, there are another 15 identified QTLs near this locus, including *QYr.ucw-1BL* (Cobo et al., 2018, 2019), *QYr.saas-1BL* (Yang et al., 2019), *QYr.cdl-1BL* (Kolmer et al., 2012), *QYr.tam-1B* (Basnet et al., 2014), *QYr.cim-1BL* (Lan et al., 2014; Calvo-Salazar et al., 2015; Ren et al., 2017; Ponce-Molina et al., 2018b), *QPm.caas-1BL.1* (Jia et al., 2018), *QYrdr.wgp-1BL.2* (Hou et al., 2015), *QYr.hebau-1BL* (Zhang et al., 2019b; Gebrewahid et al., 2020), *QPm.vt-1B* (Liu et al., 2001; Tucker et al., 2007), *QPst.jic-1B* (Melichar et al., 2008), *QYrex.wgp-1BL* (Lin and Chen, 2009), *QYr.sun-1B* (Bariana et al., 2010; Zwart et al., 2010; Bansal et al., 2014), *QYrsn.nwafu-1BL* (Huang et al., 2021), *QYrqin.nwafu-1BL* (Zeng et al., 2019), and *QYr.nwafu-1BL.3* (Liu et al., 2023). Ten of them were co-localized with *QPm/Yr.caas-1B* detected in this study.

Cobo et al. (2019) mapped locus *QYr.ucw-1BL* within a genetic interval of 0.06 cM flanking by molecular markers *ucw.k34* and *ucw.k18*. The physical location of *QYr.ucw-1BL* in the Chinese Spring reference genome was 115 kb (670.03–670.14 Mb). The RLK-encoding gene *TraesCS1B01G454100* is the high-confidence gene annotated in this genomic interval only. However, KASP markers *ucw.k34* and *ucw.k18* detected no polymorphism between Qingxinmai and line 041133, and no variation in DNA sequence was observed in the ORF coding region at *TraesCS1B01G454100* between the two parental lines. These results indicate that *TraesCS1B01G454100* is most likely not the candidate gene of *QPm/Yr.caas-1B*.

### Comparison of *QYr.caas-3B* and *QYr.caas-6B.1* with known QTLs

*QYr.caas-3B* and *QYr.caas-6B.2* for stripe rust resistance were stably detected on chromosomes 3BS and 6BL of line 041133. There exist 12 reported QTLs conferring APR and four genes for ASR against stripe rust on 3BS. Eight of them, including *QYr.ucw-3BS* in UC1110 (Lan et al., 2017), *QYr.tam-3B* in Quaiu (Basnet et al., 2014), *QYr.cim-3BS.2* in Frankolin (Lan et al., 2014), *QYr.inra-3BS* in Renan (Dedryver et al., 2009), *QYr.ar-3BS* in VA96W-270 (Subramanian et al., 2016), *QYr.nwafu-3BS* in FDC12 (Liu et al., 2022), *QYr‐3B.1* in Pavon76 (William et al., 2006), and *QYr.ucw‐3B.2* in IWA5202 (Maccaferri et al., 2015), overlapped with *QYr.caas-3B* detected in the current study. The interval of *QYr.caas-3B* in the Chinese Spring reference genome sequence is 510 kb (6.25–6.76 Mb), which is similar to locus *Sr2/Lr27/Yr30* (Spielmeyer et al., 2003). Therefore, *QYr.caas-3B* is likely *Yr30*.

Nine loci conferring stripe rust resistance have been detected on the chromosome arm 6BL. They included *QYrpav.cim-6BL* in Pavon 76 (William et al., 2006), *QYr.inra-6B* in Renan (Dedryver et al., 2009), *QYr.caas-6BL* in Pingyuan 50 (Lan et al., 2010a), *QYr.cim-6BL* in Pastor (Rosewarne et al., 2012), *QYrdr.wgp-6BL.2* in Druchamp (Hou et al., 2015), *QYr.wsu-6BL* in IWA7257 (Bulli et al., 2016), *QYr.nwafu-6BL.1* in Fried (Wu et al., 2018), *QYr.nwafu-6BL.2* in P10078 (Zeng et al., 2019), and *QYr.nwafu-6BL.3* in Xinong1376 (Mu et al., 2019). The physical location of *QYr.caas-6BL.2* in the Chinese Spring reference genome is 230 kb (576.02–576.25 Mb). It coincides with *QYr.inra-6B* and *QYr.wsu-6BL* but differs from the other loci previously reported on this chromosome arm.

### Pyramiding QTLs for resistance to powdery and stripe rust

Genes or QTLs performing APR usually last a long period of time. For example, locus *Lr46*/*Yr29* has been used in breeding and remained effective against stripe rust and leaf rust for several decades in different countries, such as Mexico (Li et al., 2014; Lan et al., 2015), India (Ponce-Molina et al., 2018a), Uruguay (Kohli, 1986), and Argentina (Kolmer, 2015; Cobo et al., 2018). Wheat breeding line 041133 has maintained its stripe rust for approximately two decades since it was developed in Qinghai Province (L. Ma, unpublished data). We confirmed that it was resistant to stripe rust, as well as powdery mildew, at the adult-plant stage throughout the course of this study from 2019 to 2023 in all the field tests. As the shift in virulence patterns of *Pst* races, virulent pathogen pathotypes may accumulate, resulting in the defeat of the disease resistance loci. Pyramiding different loci for resistance to the same disease or even different diseases can mitigate the risk of ineffectiveness for resistance loci. We have developed a KASP marker *KASP_1B_668028290* for tracing *QPm/Yr.caas-1B* in molecular marker-assisted selection. We stacked six loci for resistance to powdery mildew and stripe rust in four breeding lines. These lines demonstrate excellent disease resistance and will be useful as a new source of disease resistance in wheat breeding.

## Data availability statement

The original contributions presented in the study are included in the article/**Supplementary Material**. Further inquiries can be directed to the corresponding authors.

## Author contributions

YL: Data curation, Methodology, Software, Writing – original draft, Writing – review & editing. JH: Data curation, Methodology, Software, Writing – review & editing. HLL: Data curation, Writing – review & editing. DQ: Data curation, Methodology, Writing – review & editing. YQ: Data curation, Writing – review & editing. JD: Data curation, Writing – review & editing. LH: Data curation, Writing – review & editing. LM: Data curation, Writing – review & editing. QW: Methodology, Writing – review & editing, Funding acquisition, Software, Supervision. ZL: Conceptualization, Funding acquisition, Supervision, Writing – review & editing. YZ: Funding acquisition, Supervision, Writing – review & editing. HJL: Funding acquisition, Supervision, Writing – review & editing, Writing – original draft.

## Supplementary material

Supplementary Figure 1 Molecular mapping of *QPm.caas-2B.1* for powdery mildew resistance at the adult-plant stage. **(A)** *QPm.caas-2B.1* for powdery mildew in the RIL population of Qingxinmai × 041133 at the logarithm of the odd (LOD) of 2.5. **(B)** Linkage map of chromosome 2B constructed using SNP markers generated by the 16K GBTS SNP array. **(C)** Comparison of the QTL (red bar) identified in this study and the genes/QTL (green bars) for resistance to powdery mildew previously mapped on chromosome 2BL based on the physical positions of linked molecular markers projected in the Chinese Spring reference genome RefSeq v1.0.

Supplementary Figure 2 Molecular mapping of *QYr.caas-3B* for stripe rust resistance at the adult-plant stage. **(A)** *QYr.caas-3B* for stripe rust in the RIL populations at the logarithm of the odd (LOD) of 3.0. **(B)** Linkage map of chromosome 3B constructed using SNP markers generated by the 16K GBTS SNP array. **(C)** Comparison of *QYr.caas-3B* (red bar) identified in this study and genes/QTL (green bars) previously mapped on chromosome 3BS for resistance to stripe rust based on the physical positions of linked molecular markers projected in the Chinese Spring reference genome RefSeq v1.0.

Supplementary Figure 3 Molecular mapping of *QYr.caas-6B.2* for stripe rust resistance at the adult-plant stage. **(A)** *QYr.caas-6B.2* for stripe rust in the RIL populations at the logarithm of the odd (LOD) of 3.0. **(B)** Linkage map of chromosome 6B constructed using SNP markers generated by the 16K GBTS SNP array. **(C)** Comparison of *QYr.caas-6B.2* (red bar) identified in this study and genes/QTL (green bars) previously mapped on chromosome 6BL for resistance to stripe rust based on the physical positions of linked molecular markers projected in the Chinese Spring reference genome RefSeq v1.0.

Supplementary Figure 4 Scatter plots of the RILs from cross Qingxinmai × 041133 for the Kompetitive allele-specific PCR (KASP) assay using the *QPm/Yr.caas-1B* linked marker *KASP_1B_668028290*. The blue and red dots represent the RILs with the CC (Qingxinmai) and AA (041133) genotypes, respectively. The non-template control is indicated by the black dots.

Supplementary Figure 5 Box plot of MDS values about *QPm.caas-1B* and DS values about *QYr.caas-1B* with genotypes of line 041133 and Qingxinmai in different environments. ns: not significant. ***P* < 0.01.

Supplementary Figure 6 Alignment of the predicted open reading form region sequences of gene *TraesCS1B01G454100*. The comparative genomes include 10+ genomes (Walkowiak et al., 2020), Zang1817 (Guo et al., 2020), Fielder (Sato et al., 2021), KN9204 (Shi et al., 2022), AK58 (Jia et al., 2023), Kariega (Athiyannan et al., 2022), and Renan (Aury et al., 2022).
