# Supplementary figures and images for: Mapping QTLs for adult-plant resistance to powdery mildew and stripe rust using a recombinant inbred line population derived from cross Qingxinmai × 041133
Source: Front Plant Sci. 2024 May 8;15:1397274. doi: 10.3389/fpls.2024.1397274 (PMC11109386; doi:10.3389/fpls.2024.1397274)

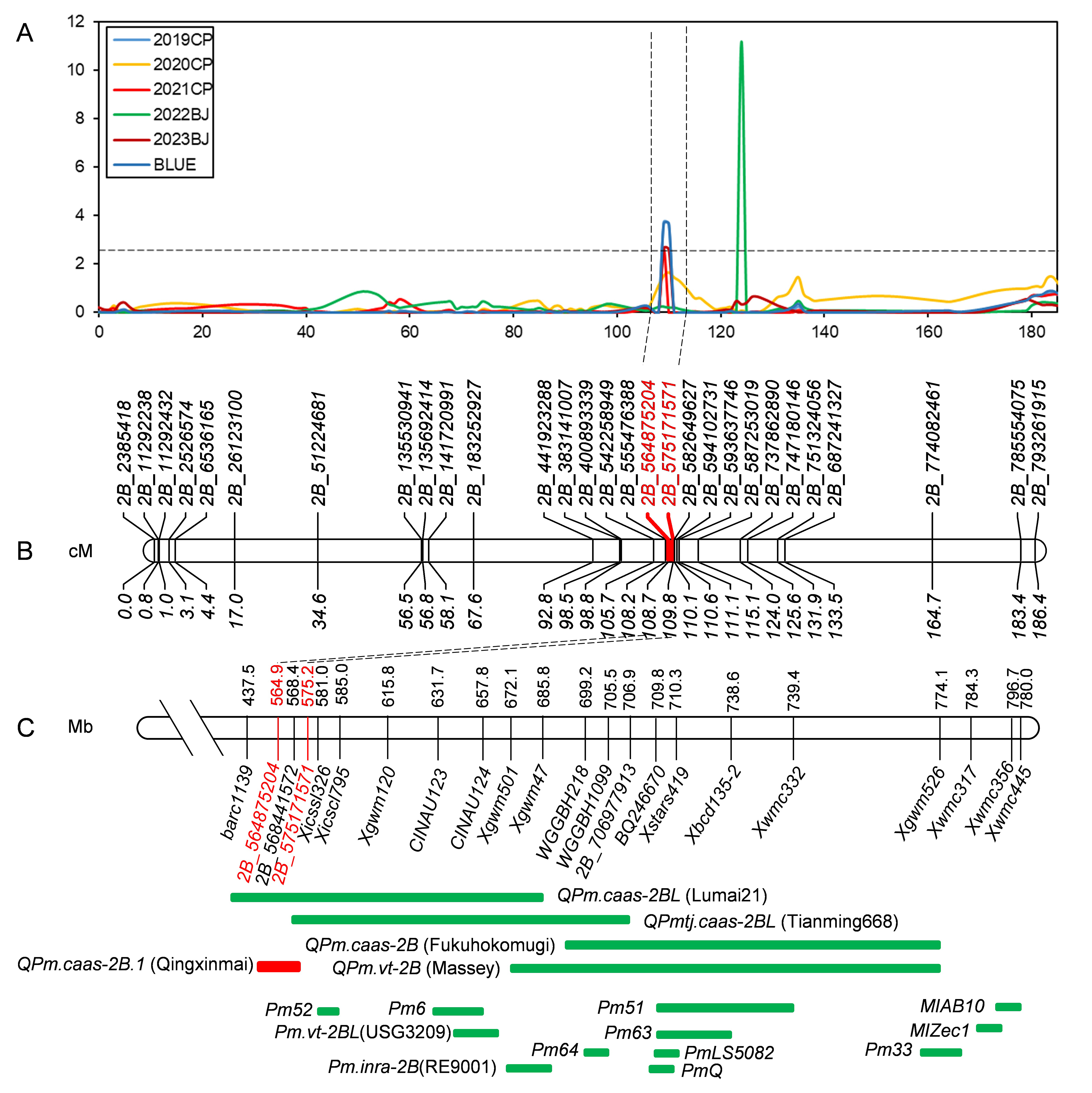

Supplement: Supplementary Figure 1 — Molecular mapping of QPm.caas-2B.1 for powdery mildew resistance at the adult-plant stage. (A) QPm.caas-2B.1 for powdery mildew in the RIL population of Qingxinmai × 041133 at the logarithm of the odd (LOD) of 2.5. (B) Linkage map of chromosome 2B constructed using SNP markers generated by the 16K GBTS SNP array. (C) Comparison of the QTL (red bar) identified in this study and the genes/QTL (green bars) for resistance to powdery mildew previously mapped on chromosome 2BL based on the physical positions of linked molecular markers projected in the Chinese Spring reference genome RefSeq v1.0. [file Image_1.jpeg]

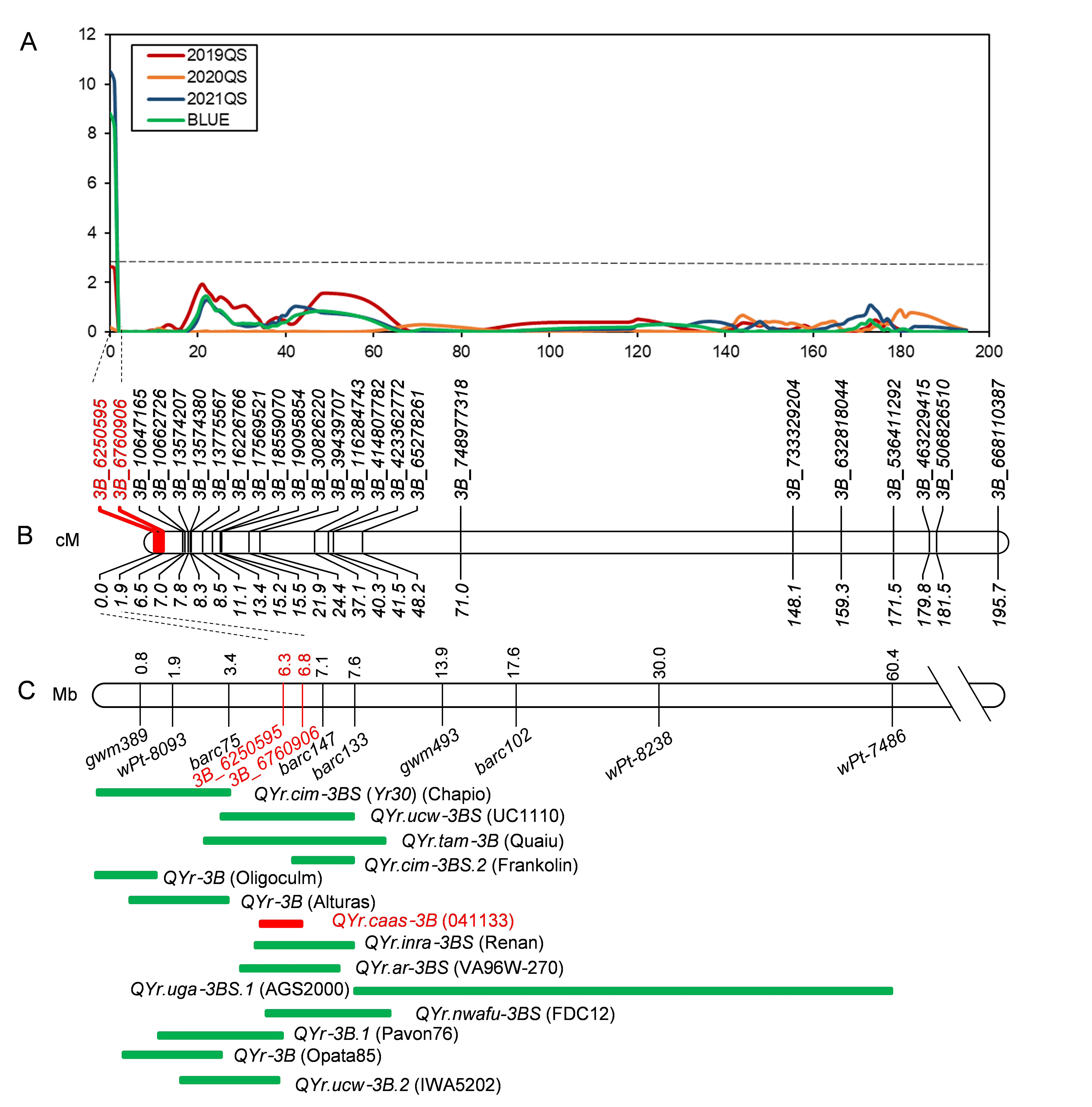

Supplement: Supplementary Figure 2 — Molecular mapping of QYr.caas-3B for stripe rust resistance at the adult-plant stage. (A) QYr.caas-3B for stripe rust in the RIL populations at the logarithm of the odd (LOD) of 3.0. (B) Linkage map of chromosome 3B constructed using SNP markers generated by the 16K GBTS SNP array. (C) Comparison of QYr.caas-3B (red bar) identified in this study and genes/QTL (green bars) previously mapped on chromosome 3BS for resistance to stripe rust based on the physical positions of linked molecular markers projected in the Chinese Spring reference genome RefSeq v1.0. [file Image_2.jpeg]

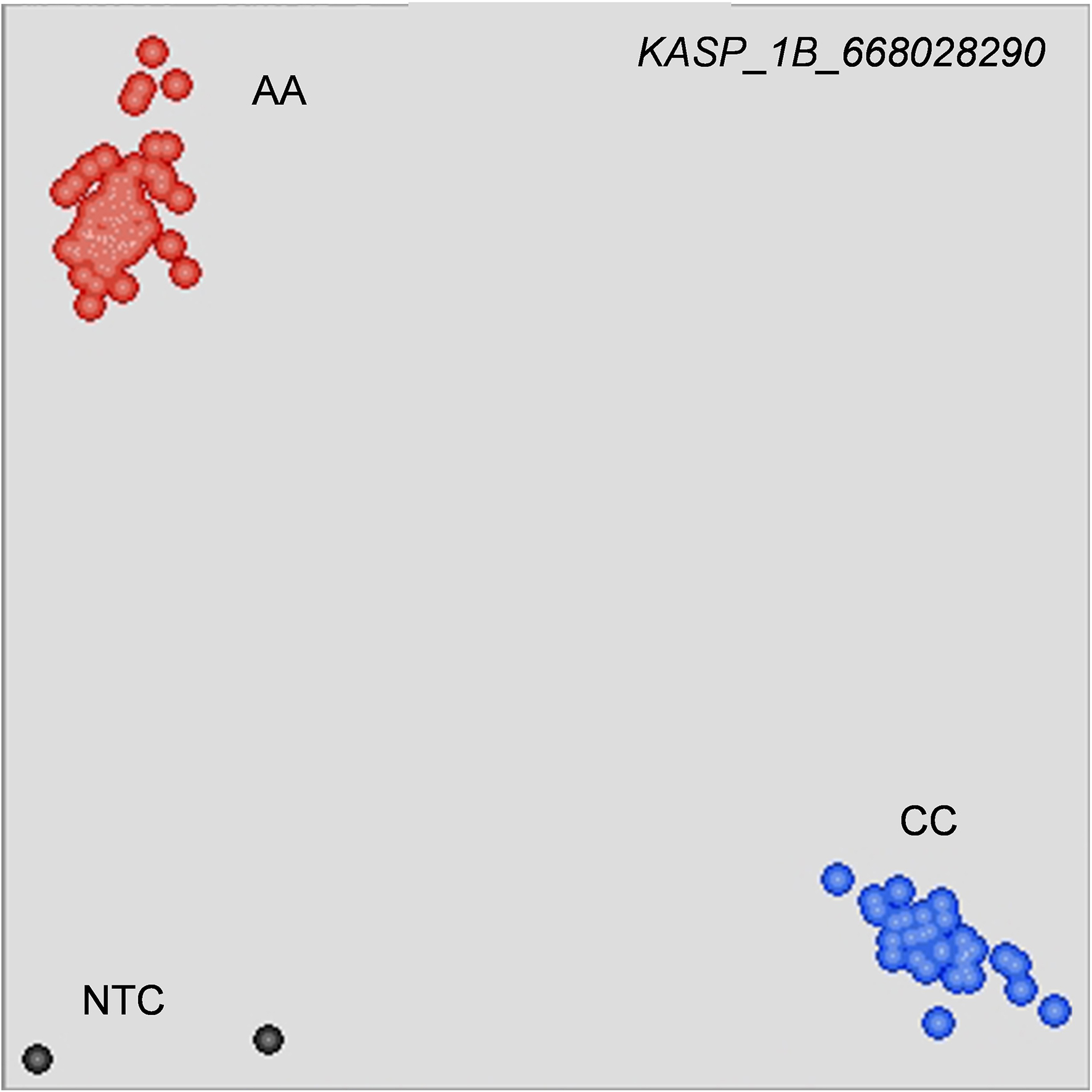

Supplement: Supplementary Figure 4 — Scatter plots of the RILs from cross Qingxinmai × 041133 for the Kompetitive allele-specific PCR (KASP) assay using the QPm/Yr.caas-1B linked marker KASP_1B_668028290. The blue and red dots represent the RILs with the CC (Qingxinmai) and AA (041133) genotypes, respectively. The non-template control is indicated by the black dots. [file Image_4.jpeg]

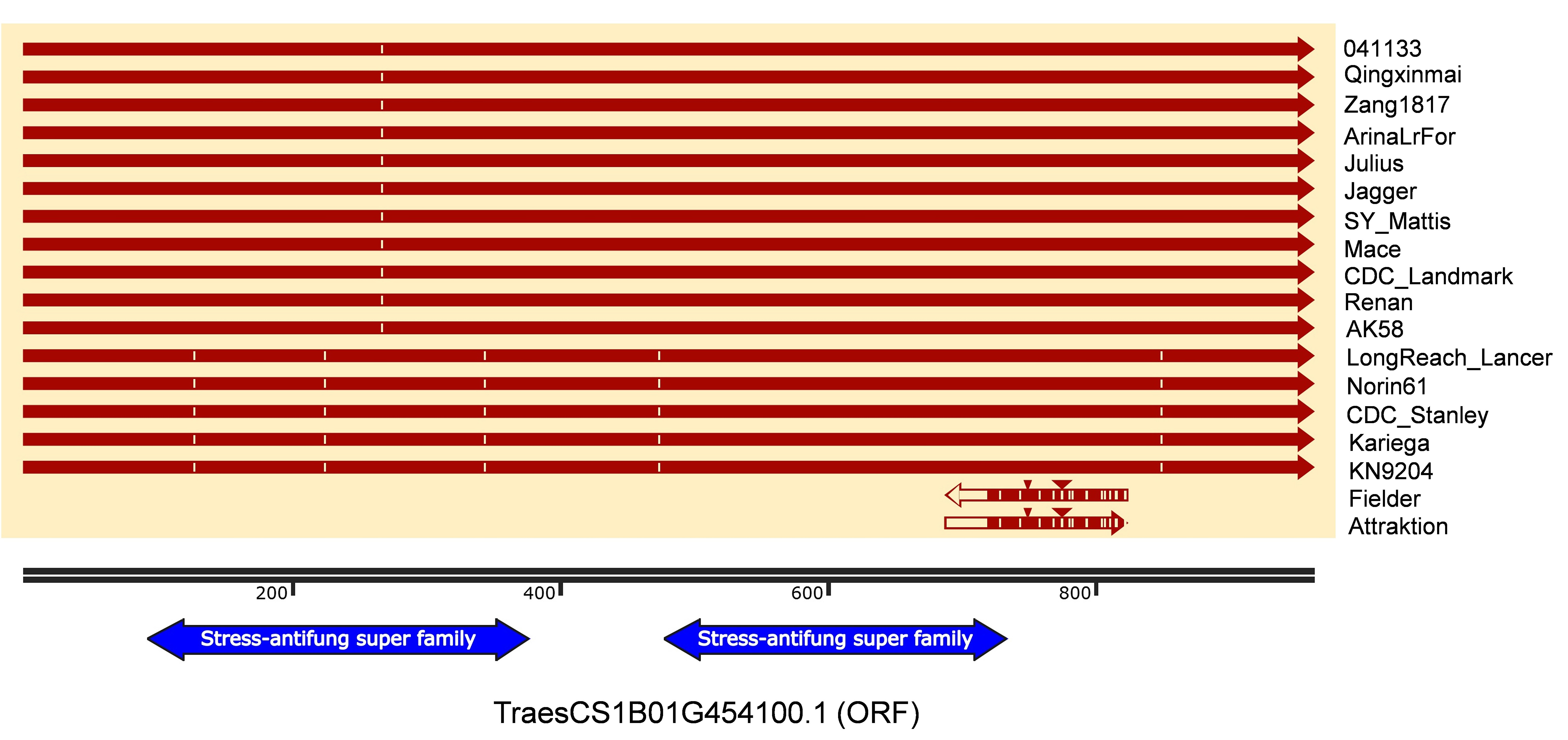

Supplement: Supplementary Figure 6 — Alignment of the predicted open reading form region sequences of gene TraesCS1B01G454100. The comparative genomes include 10+ genomes (Walkowiak et al., 2020), Zang1817 (Guo et al., 2020), Fielder (Sato et al., 2021), KN9204 (Shi et al., 2022), AK58 (Jia et al., 2023), Kariega (Athiyannan et al., 2022), and Renan (Aury et al., 2022). [file Image_6.jpeg]
